# The substance P/ Neurokinin-1 receptor signaling drives perineural invasion in pancreatic ductal adenocarcinoma (PDAC)

**DOI:** 10.3389/fcell.2026.1876023

**Published:** 2026-08-19

**Authors:** Katja Schiedlauske, Sandra Biskup, Lina Hofer, Alina Deipenbrock, Kaan Çifcibaşı, Ihsan Ekin Demir, Irene Esposito, Dirk Weyhe, Nicole Elisabeth Teusch

**Affiliations:** 1 Institute of Pharmaceutical Biology and Biotechnology, Heinrich Heine University Düsseldorf, Düsseldorf, Germany; 2 Institute of Pathology, University Hospital and Heinrich-Heine-University, Düsseldorf, Germany; 3 Department of Medicine III, TUM University Hospital, Klinikum Rechts der Isar, School of Medicine and Health, Technical University of Munich, Munich, Germany; 4 Department of Surgery, TUM University Hospital, Klinikum Rechts der Isar, School of Medicine and Health, Technical University of Munich, Munich, Germany; 5 Carl von Ossietzky Universität Oldenburg, University Hospital for Visceral Surgery, PIUS-Hospital Oldenburg, Oldenburg, Germany

**Keywords:** aprepitant, neurokinin-1 receptor, pancreatic cancer, perineural invasion, schwann cells, sensory neurons, substance P, tumor-neural niche

## Abstract

**Background:**

Pancreatic ductal adenocarcinoma (PDAC) is one of the deadliest malignancies, characterized by early metastasis, profound therapy resistance, and the highest prevalence of perineural invasion (PNI) among solid tumors. PNI fosters neuropathic pain and recurrence, ultimately correlating with poor survival. Sensory neurons promote PNI via neuropeptidergic signaling, including substance P (SP), while Schwann cells undergo injury-like reprogramming (GFAP^+^/p75NTR^high^), facilitating tumor invasion through chemoattractant secretion. Although the activation of neurokinin-1 receptor (NK-1R) via SP is implicated in tumor invasion and pain signaling, its role in coordinating bidirectional crosstalk within the PDAC neural niche remains elusive. In this study, we investigated the SP/NK-1R axis as a mediator of bidirectional crosstalk between tumor cells, sensory neurons, and Schwann cells in pancreatic cancer.

**Methods:**

To elucidate the role of the SP/NK-1R axis in perineural invasion, we employed a series of complementary *in vitro* approaches. First, NK-1R expression was assessed by immunofluorescence in both primary PDAC tissue slices and PDAC tumor cell lines. To model the tumor-neural niche, MiaPaCa-2 cells were exposed to conditioned medium derived from iPSC-derived sensory neurons and primary human Schwann cells. Furthermore, to investigate the functional consequences of this signaling axis, we established a co-culture system composed of MiaPaCa-2 cells and Schwann cells, in which the number of invaded MiaPaCa-2 cells was quantified. Axon-guided tumor invasion was further examined using the OrganoPlate® Graft platform, where MiaPaCa-2 spheroids were co-cultured with sensory neurons. Moreover, NK-1R was pharmacologically inhibited using the FDA-approved antagonist aprepitant, and its effect on MiaPaCa-2 cell invasion was evaluated in both co-culture settings. Finally, the potential for therapeutic synergy was explored by combining aprepitant with the chemotherapeutic agent paclitaxel to assess potential synergy on cytotoxicity.

**Results:**

Immunofluorescence analysis of MiaPaCa-2, patient-derived tumor cells and primary PDAC tissue slices, revealed high NK-1R protein expression, establishing NK-1R as a putative clinically relevant target in PDAC. To model the bidirectional crosstalk within the PDAC niche, we examined NK-1R regulation under co-culture conditions. Conditioned media from sensory neurons and primary human Schwann cells significantly upregulated NK-1R expression in the MiaPaCa-2 cells. Reciprocally, exposure of both sensory neurons or Schwann cells to MiaPaCa-2 cells conditioned medium induced NK-1R upregulation, confirming bidirectional signaling. Consistent with this, supernatant from MiaPaCa-2/sensory neuron or MiaPaCa-2/Schwann cells co-cultures demonstrated a substantial increase in SP levels, indicating active neuropeptidergic communication within the tumor-neural niche. To assess the functional consequences of this crosstalk on tumor invasiveness, we employed our co-culture invasion models. Schwann cells significantly increased the number of invaded MiaPaCa-2 cells in co-culture. Furthermore, using the OrganoPlate® Graft platform, co-culture of MiaPaCa-2 spheroids and iPSC-derived sensory neurons demonstrated pronounced axon-guided tumor, validating the human 3D PNI model. Finally, to evaluate the therapeutic potential of NK-1R inhibition, we treated co-cultures with the FDA-approved NK-1R antagonist aprepitant. Pharmacological NK-1R blockade significantly reduced MiaPaCa-2 cell invasion in both sensory neuron and Schwann cell co-cultures. Moreover, combining aprepitant with paclitaxel synergistically enhanced cytotoxicity, revealing a dual role for NK-1R in driving both invasion and chemoresistance.

**Conclusion:**

Collectively, our findings confirm and extend previous work by implicating SP/NK-1R axis as a potential driver of perineural invasion in PDAC. By leveraging a human 3D microfluidic co-culture PNI model, we provide evidence that NK-1R signaling contributes to sustaining bidirectional tumor-neural crosstalk and promoting invasive progression. Critically, pharmacological inhibition of NK-1R using the FDA-approved antagonist aprepitant not only suppressed perineural invasion but also synergistically enhanced paclitaxel-induced cytotoxicity, highlighting dual vulnerability in both neural invasion and chemoresistance. Together, these findings position SP/NK-1R as a target, offering a novel and translationally relevant strategy to simultaneously disrupt tumor-nerve interactions and improve chemotherapy response in PDAC.

## 1 Introduction

Pancreatic ductal adenocarcinoma (PDAC) is among the most lethal malignancies, with a 5-year survival rate of 13% (Siegel et al., 2025; Siegel et al., 2024). PDAC is characterized by early metastasis and intrinsic therapy resistance (Costamagna et al., 2025; Hartupee et al., 2024). Combination chemotherapy with FOLFIRINOX (5-fluorouracil, leucovorin, irinotecan, and oxaliplatin) or gemcitabine plus albumin-bound (nab-) paclitaxel represents the standard first-line treatment (Di Costanzo et al., 2023), while gemcitabine plus nab-paclitaxel is frequently employed in patients who are unable to tolerate FOLFIRINOX (Orlandi et al., 2024). The tumor microenvironment (TME) of PDAC is defined by a dense desmoplastic stroma dominated by cancer-associated fibroblasts (CAFs), which markedly increase intratumoral pressure, resulting in vascular compression and vessel collapse (Zhang X. et al., 2025). In parallel, the TME is profoundly immunosuppressive, enriched in regulatory T-cells, tumor-associated macrophages and myeloid-derived suppressor cells, promoting immune evasion and therapy resistance (Pan et al., 2025). In addition to stromal and immune components, nerves constitute a functionally relevant component of the TME. They contribute to perineural invasion (PNI), a pathological hallmark of PDAC histopathologically found in nearly 100% of cases (Lovecek et al., 2025; Deng et al., 2025). PNI is characterized by tumor cell invasion of neural structures along the epineural, perineural and endoneural layers, which creates a tumor-neural niche within the TME (Selvaggi et al., 2025). PNI is strongly associated with cancer-associated pain driven by neuroinflammation and nerve injury (Catalano et al., 2025; Giri et al., 2024). Moreover, PNI correlates with a higher risk of local recurrence after surgical resection (Crippa et al., 2022; Schouten et al., 2025). Clinically, PNI is an important prognostic factor correlating with reduced overall and disease-free survival (Ni et al., 2023; Meng et al., 2026). Increasing evidence suggests that PNI is a neurobiologically driven process in which tumor cells promote nerve growth within the TME. Conversely, nerves recruit tumor cells via chemotactic signaling. These bidirectional interactions shape a distinct tumor-neural niche that sustains PNI (Kane et al., 2026; Chen et al., 2019). However, the precise mechanisms remain incompletely understood.

Within this context, Deborde et al. have described that Schwann cells, the predominant glial cells of the peripheral nervous system, display remarkable plasticity and undergo tumor-associated cellular reprogramming, increasing the invasion of tumor cells toward the tumor-neural niche (Zhang Y. et al., 2025; Ebrahim et al., 2025; Deborde et al., 2016). Schwann cells dedifferentiate into a non-myelinating, tumor-associated GFAP^+^/p75NTR^high^ phenotype, reminiscent of the repair Schwann cell state observed during nerve injury or infections (Deborde et al., 2022; Cai et al., 2024). GFAP^+^/p75NTR^high^ Schwann cells adopt a highly secretory profile, marked by the release of pro-invasive mediators, including neurotrophic factors such as nerve growth factor (NGF), brain-derived neurotrophic factor (BDNF), glial cell line-derived neurotrophic factor (GDNF), and neurotrophin-3 (NT-3), as well as chemokines (C-C motif chemokine ligand 2 (CCL2), CCL7)), creating a pro-invasive milieu (Deborde et al., 2016; Deborde et al., 2022; Bahmad et al., 2023; Weitz et al., 2023). Furthermore, Schwann cells actively shape the extracellular matrix (ECM) within the TME through the secretion of ECM-remodeling enzymes, including matrix metalloproteinase-2 (MMP-2), thereby promoting tumor cell invasion (Zhang et al., 2022; Rangel-Sosa et al., 2024; He et al., 2025; Demir et al., 2017; Gregory et al., 2025; Tian et al., 2022; Ferdoushi et al., 2020). Enhanced adhesion molecule expression including L1 cell adhesion molecule (L1CAM) and neural cell adhesion molecule-1 (NCAM1) expressed by Schwann cells and tumor cells drives the formation of direct cellular tracks, providing structural guidance for tumor cell dissemination (Deborde et al., 2016; Na’ara et al., 2019; Deborde and Wong, 2022). Although Schwann cells critically promote PNI, the role of peripheral nerves remain less clearly defined (Chen et al., 2019; Fu et al., 2026). Intratumoral peripheral nerves undergo pronounced remodeling during tumor progression, marked by increased size of intrapancreatic nerves (neural hypertrophy) and elevated neural density (Xue et al., 2023; Sur et al., 2025; Thiel et al., 2025). In this context, sensory neurons have been implicated in promoting tumor progression (Ren et al., 2025). Notably, intratumoral sensory neurons actively attract tumor cells through neuropeptidergic signaling (Giri et al., 2024; Mardelle et al., 2024). In mouse dorsal root ganglia (DRGs) and preclinical murine cancer models neuropeptides such as calcitonin gene-related peptide (CGRP) and substance P (SP) exert direct protumorigenic effects by activating oncogenic signaling cascades in tumor cells, including MAPK/ERK, PI3K/Akt, and NF-κB pathways (Thiel et al., 2025; Chen et al., 2023; Huang et al., 2018). Through these mechanisms, they contribute to epithelial–mesenchymal transition (EMT), thereby promoting tumor dissemination (Banh et al., 2020). Furthermore, Wang et al. showed that CCL2 secreted by mouse DRGs enhances tumor cell invasion by inducing cytoskeletal reorganization via activation of the paxillin signaling axis (Wang et al., 2023). In conclusion, whether axon-guidance mechanisms directly guide tumor cell migration remains to be determined, and the precise role of sensory neurons in PNI is largely unexplored.

The SP/neurokinin-1 receptor (NK-1R) axis is well-established in the pathophysiology of neuropathic pain, including cancer-associated pain (Li et al., 2013a; Esteban et al., 2006). SP released from sensory neurons binds to its high-affinity receptor, NK-1R, on neurons of the dorsal horn and non-neuronal cells, including dendritic cells and macrophages, leading to neurogenic inflammation and pain (Lambrecht, 2001). Emerging evidence highlights the SP/NK-1R axis as a critical mediator of tumor–nerve interactions. Notably, in various solid cancers, including breast, colorectal, prostate and head and neck cancer, SP and NK-1R are overexpressed, furthermore, high levels of NK-1R correlate with poor outcomes (Al-Keilani et al., 2023; Zhang et al., 2023; Chen et al., 2016; Friess et al., 2003a; Muñoz et al., 2012; Capodanno and Hirth, 2023; Padmanaban et al., 2024). In colorectal cancer, elevated SP and NK-1R expression were significantly associated with lymph node metastasis, advanced tumor stage, and reduced survival (Chen et al., 2016). A systematic meta-analysis of head and neck carcinogenesis demonstrated significant overexpression of SP and NK-1R in malignant lesions compared to benign tissue, highlighting the role of the SP/NK-1R axis in tumor development and its potential as a prognostic marker (González-Moles et al., 2021). In PDAC, NK-1R expression is upregulated in tumor lesions compared to healthy pancreatic tissue, particularly in advanced stages (Friess et al., 2003a). Upon SP release from sensory neurons, NK-1R signaling in PDAC cells drives EMT and ECM remodeling via the upregulation of MMP-2 (Huang et al., 2018; Li et al., 2013b). Additionally, NK-1R activation enhances tumor cell proliferation and confers resistance to apoptosis through the activation of the MAPK/ERK, PI3K/Akt, NF-κB, and STAT3 signaling pathways (Beirith et al., 2021a; Ji et al., 2022a). Furthermore, a preclinical PDAC mouse model demonstrated that sensory denervation reduced NK-1R expression in pancreatic intraepithelial neoplasms (PanIN), thereby attenuating PanIN progression (Sinha et al., 2017). Aprepitant, an FDA-approved NK-1R antagonist used to manage chemotherapy-induced nausea, has attracted growing interest for its anticancer potential (Muñoz and Rosso, 2010; Muñoz and Coveñas, 2014). *In vitro* and *in vivo* studies across multiple solid tumors including pancreatic cancer have demonstrated that aprepitant inhibits tumor cell proliferation and induces apoptosis, and may further contribute to overcoming chemoresistance (Friess et al., 2003a; Muñoz et al., 2012; Beirith et al., 2021b; Engür-Öztürk et al., 2025). However, the potential synergistic effect of aprepitant with first-line regimens has not yet been investigated.

In particular, paclitaxel, a microtubule-stabilizing agent and a first-line regimen for PDAC, is strongly associated with chemotherapy-induced neuropathic pain (Kawashiri et al., 2021). Clinically, 30% of patients treated with paclitaxel develop neuropathic pain, which frequently necessitates dose reduction or the discontinuation of chemotherapy (Xu et al., 2022). Preclinical mouse models have demonstrated sustained SP release following paclitaxel treatment, suggesting a putative role of SP/NK-1R signaling (Tatsushima et al., 2011). To date, there is a lack of studies evaluating clinical outcomes in PDAC patients, including behavioral outcomes and potential synergistic effects, when treated with paclitaxel in combination with aprepitant. Here, we demonstrate that NK-1R expression in tumor cells is induced by both Schwann cells and sensory neurons, while tumor cells reciprocally elicit NK-1R expression in these neural cell types, thereby sustaining bidirectional tumor–neural crosstalk. By leveraging a novel human 3D microfluidic co-culture model, we provide the first observation of axon-guided tumor cell migration in a fully human system. Pharmacological inhibition of NK-1R with aprepitant significantly reduced axon-guided tumor cells invasion, indicating a functional contribution of NK-1R signaling in driving PNI. Notably, the combination of aprepitant with paclitaxel enhanced the cytotoxic efficacy in PDAC cells. Together, these findings highlight the SP/NK-1R pathway as a translational therapeutic target capable of disrupting tumor–nerve interactions and potentially enhancing the chemotherapeutic response in PDAC patients.

## 2 Materials and methods

### 2.1 Cell line

MiaPaCa-2 cell line, obtained from the American Type Culture Collection (#CRL-1420, ATCC, Virginia, United States), were kindly provided by the Institute of Pathology (University Hospital and Heinrich Heine University Düsseldorf). MiaPaCa-2 cells were cultured in Roswell Park Memorial Institute Medium (RPMI) (#22400089, Gibco, NY, United States) supplemented with 10% fetal bovine serum (FBS) (#10270–106, Gibco), 2,5% horse serum (HS) (#26050088, Gibco) and 1% penicillin–streptomycin (#15140122, Gibco). Cells were incubated under aseptic conditions at 37 °C in a humidified atmosphere supplemented with 5% CO_2_.

### 2.2 Spheroid formation

MiaPaCa-2 spheroids were generated according to our previously described protocol reported in Deipenbrock et al., 2025 (Beirith et al., 2021a). Briefly, MiaPaCa-2 cells were seeded with a concentration of 3 × 10^4^ cell/mL in culture medium supplemented with 2,5% Matrigel (#356230, Corning, NY, United States) in an ultra-low attachment (ULA) coated surface plates (#650970, Greiner Bio-One, Kremsmünster, Austria) and centrifuged for 5 min at 1500 rpm. After 24 h 40 µL of culture medium was added. Spheroids were used for downstream assay at day 4.

### 2.3 Primary human Schwann cells (hSC)

Primary human Schwann cells (hSC), sourced from ScienCell (#1700, ScienCell, Carlsbad, United States), were kindly provided by the Department of Surgery (Klinikum rechts der Isar, Technical University of Munich). hSC were cultured according to the provided protocol using complete Schwann cell medium (#1701, ScienCell) at 37 °C in a humidified atmosphere with 5% CO_2_ supplementation.

### 2.4 Primary pancreatic cancer cells

PDAC tumor tissue was obtained during resection at Pius-Hospital Oldenburg, Germany. Clinicopathological characteristics of the included PDAC patients are summarized in Supplementary Table S1. All patients underwent surgical resection without neoadjuvant treatment. Fresh PDAC tumor tissue was meticulously minced into segments measuring less than 1 cm^3^. Tissue segments were subjected to an enzymatic digestion process using a solution consisting of 0.6 mg/mL collagenase IV (#17104–019, Gibco), 0.1 mg/mL DNase (#10104159001, Roche, Basel, Switzerland), and 2% FBS in RPMI and incubated at 37 °C with continuous agitation for 60 min. Cell suspension was passed through a 70 μm cell strainer with washing steps using PBS supplemented with 2% FBS. After centrifugation at 550 *g* for 7 min, cell pellet was resuspended in DMEM/F12 (#11320074, Gibco) with 20% FBS. The establishment of pure tumor cell lines was accomplished through the implementation of differential trypsinization, a method previously delineated by Chai et al., in 2024 (Chai et al., 2024).

### 2.5 Human PDAC slices

Human PDAC slices were kindly generated at the Institute of Pathology from the University Hospital Düsseldorf. All samples were anonymized and their use was approved by the ethics committee of the Heinrich Heine University Düsseldorf (study number 2022–2170). Subsequently, slices were formalin-fixed and paraffin-embedded (FFPE) according to standard histopathological procedures. 1.5 µm-thick sections were prepared using a microtome and mounted onto glass. For immunofluorescence analysis, slices were initially deparaffinized in xylene (#447240010, Thermo Scientific, Waltham, United States) followed by rehydration through a series of graded ethanol dilutions (100%, 98% and 70%) and rinsing in distilled water. Antigen retrieval was performed by heat-induced antigen retrieval in Tris-EDTA buffer, pH 9 (#1052.1, Carl Roth, Karlsruhe, Germany). Following antigen retrieval, slices were processed according to the immunofluorescence staining protocol used for 2D cultures.

### 2.6 PDAC tumor samples

PDAC tumor tissue was obtained during resection at Pius-Hospital Oldenburg, Germany. The use of human tissue samples was approved by the ethics committee at Carl von Ossietzky University Oldenburg and confirmed by Heinrich Heine University Düsseldorf (study number 2022–058). All patients provided written informed consent prior to tissue collection. Clinicopathological characteristics of the included PDAC patients are summarized in Supplementary Table S1. All patients underwent surgical resection without neoadjuvant treatment. Fresh PDAC tissue was collected and cylindrical tissue cores with a diameter of 5 mm were generated using a sterile biopsy punch (#15115–11, Rapid Core-Punch, Integra LifeSciences, Plainsboro, United States). PDAC tumor explants were generated by sectioning the 5 mm tissue cores into 250 µm-thick slices using a tissue slicer (#MD6000, Alabama R&D, Munford, United States) equipped with an oscillating blade (PERS74-002–0000, Personna Medical, Eau Claire, United States) in accordance with the manufacturer’s instructions. Immediately after sectioning, samples were transferred to 4% paraformaldehyde (PFA) and fixed for 30 min at room temperature. Following fixation, samples were further processed for 3D immunofluorescence staining.

### 2.7 Differentiation of iPSC-derived sensory neurons (iPSC-derived SN)

Human induced pluripotent stem cells (iPSC) were obtained from ALSTEM (#iPSC11, Richmond, United States). iPSCs were cultured on Matrigel (#354277, Corning) coated 6-well plates in 2 mL complete mTeSR plus medium (#100–0276, STEMCELL Technologies, Vancouver, Canada) at 37 °C in a humidified atmosphere supplemented with 5% CO_2_ with full medium change every 2 days. A scoring check was performed daily in accordance with the methodology proposed by Tigges et al. (Tigges et al., 2021). For differentiation, iPSCs were used from passages 3–7.

#### 2.7.1 Neural crest (NCC) differentiation

The iPSCs were differentiated using the STEMdiff Neural Crest Differentiation Kit (#08610, STEMCELL Technologies), following the instructions provided in the protocol. In brief, for single-cell splitting, iPSCs were detached with Gentle Cell Dissociation Reagent (#07174, STEMCELL Technologies) for 8 min at 37 °C and resuspended in STEMdiff Neural Crest Differentiation Medium, supplemented with 10 µM Y-27632 (#Y0503, Sigma-Aldrich, St. Louis, United States). A total of 10^6^ cells were seeded per well in a 6-well plate (#83.3920, Sarstedt) and incubated for a period of 7 days at 37 °C with 5% CO_2_ in a humidified atmosphere, with a daily medium change. NCCs were subsequently cryopreserved in CryoStor® CS10 (#07930, STEMCELL Technologies) or directly differentiated into sensory neurons.

#### 2.7.2 Sensory neuron differentiation (iPSC-derived SN)

Sensory neurons (iPSC-derived SN) were generated from intermediate neural crest cells (NCC) through a neural precursor stage, ultimately yielding mature sensory neurons. NCCs were differentiated into sensory neuron precursor cells using the STEMdiff™ Sensory Neuron Differentiation Kit (#100–0341, STEMCELL Technologies). Cells were seeded at a density of 3 × 10^5^ cells per well in Matrigel coated 24-well plates containing 500 µL of STEMdiff™ Neural Crest Differentiation Medium supplemented with 10 µM Y-27632. After 24 h, the medium was replaced with 500 µL of STEMdiff™ Sensory Neuron Differentiation Medium without Y-27632, which was refreshed daily. Following 7 days, cells were transitioned to the maturation stage using the STEMdiff™ Sensory Neuron Maturation Kit (#100–0684, STEMCELL Technologies). The maturation medium was refreshed every 2–3 days, and fully differentiated iPSC-derived SN were used between days 21 and 28.

### 2.8 Differentiation of iPSC-derived SN in the OrganoPlate® graft

Differentiation of iPSC-derived SN in the OrganoPlate® Graft was performed using iPSC-derived SN precursors at day 14 of differentiation. Prior to cell seeding, the extracellular matrix (ECM) channel was prefilled with a mixture of Matrigel® and STEMdiff™ Sensory Neuron Differentiation Medium (1:1) and allowed to polymerize at 37 °C for 10 min iPSC-derived SN precursors were mechanically detached and 2.5 × 10^5^ cells were resuspended in STEMdiff™ Sensory Neuron Differentiation Medium supplemented with 10 µM Y-27632. The cell suspension was mixed with Matrigel® (1:1) and seeded into the iPSC-derived SN channel. Following polymerization at 37 °C for 10 min, all channels were filled with STEMdiff™ Sensory Neuron Differentiation Medium containing 10 µM Y-27632. The plate was placed on an orbital rocker (7° inclination, 8-min interval) to establish bidirectional perfusion flow and incubated at 37 °C in a humidified atmosphere containing 5% CO_2_. After 24 h, the medium was replaced with STEMdiff™ Sensory Neuron Maturation Medium and subsequently refreshed every 2–3 days. After 2 weeks of maturation, differentiated iPSC-derived SN were characterized by immunostaining for the sensory neuron markers peripherin and β-III-tubulin.

#### 2.8.1 Integration of MiaPaCa-2 spheroids into the OrganoPlate® graft

MiaPaCa-2 spheroids were generated as described previously, and spheroids at day 4 of formation were used for co-culture experiments. After 7 days of iPSC-derived SN maturation in the OrganoPlate® Graft, a day-4 MiaPaCa-2 spheroid was embedded in a mixture of Matrigel® and starvation medium (1:1) and seeded into the spheroid chamber. Control chips were prepared in parallel without spheroid addition. Following polymerization at 37 °C for 10 min, all channels were filled with a mixture of sensory neuron maturation medium and starvation medium (DMEM supplemented with 0.5% FBS and 1% penicillin-streptomycin) (1:1). Medium exchange was performed every 2–3 days. Co-culture was maintained for an additional 7 days. Cells were fixed with 4% PFA and immunofluorescence labelled according to the protocol for immunofluorescence staining 3D. Number of axon-guided MiaPaCa-2 cells were captured using the CQ1 Confocal Imaging Cytometer (#90ZA00673, Yokogawa, Tokyo, Japan) and quantified with the CellPathFinder software (version 3.06.06.06, Yokogawa).

### 2.9 Conditioned medium (CM) production

MiaPaCa-2 cells were seeded at a density of 2 × 10^5^ cells/mL into a 6-well plate (#83.3920, Sarstedt). HSC were seeded at a density of 3 × 10^5^ cells/well onto a poly-l-lysine-coated 6-well plate. After 24 h, the medium was replaced with starvation medium. The medium was collected after 48 or 72 h and the supernatant was stored at −80 °C. For generation of CM of iPSC-derived SN, medium of mature neurons (day 21) was collected after 48 h of incubation.

### 2.10 Substance P ELISA

Extracellular SP concentrations were quantified using Substance P ELISA kit (#ab288318, Abcam, Cambridge, United Kingdom) following the manufacturer’s instructions. Briefly, iPSC-derived SN, hSC and MiaPaCa-2 cells were cultured under both mono- and co-culture conditions. After a 24 h incubation period, supernatant was collected and centrifuged at 1500 *g* for 10 min at 4 °C to remove cellular debris. For experiments involving paclitaxel treatment, supernatants were harvested 2 h post-treatment. Fluorescence intensity was measured using a TECAN microplate reader (Tecan Group, Männedorf, Switzerland) with excitation at 405 nm and emission detection at 570 nm as the reference wavelength.

### 2.11 Spherical invasion assay

The invasion assay was adapted from (Fangmann et al., 2018; Schiedlauske et al., 2024). In summary, MiaPaCa-2 cells and hSC were diluted in starvation medium (0,5% FBS) mixed 1:1 with Matrigel (#356230, Corning) with a cell concentration of 5 × 10^7^ cells/mL. Per cell type 6 μL cell-Matrigel solution was placed into a 24-well plate (#83.3922, Sarstedt) and allowed to polymerize for 30 min at 37 °C. An ECM bridge, composed of a 1:1 mixture of Matrigel and starvation medium was positioned between the cell drops. After 72 h cells were fixed with 4% PFA and immunofluorescent-labelled. Number of invaded MiaPaCa-2 cells were captured using the CQ1 Confocal Imaging Cytometer (Yokogawa) and quantified with the CellPathFinder software (Yokogawa).

### 2.12 Immunofluorescence staining

#### 2.12.1 2D immunofluorescence staining

Cells were fixed with 4% PFA solution for 15 min, followed by permeabilization with 0.25% Triton X-100 for 30 min. Non-specific binding of primary antibodies was prevented by incubation with 0.1% bovine serum albumin (BSA) (#9638, Carl Roth, Karlsruhe, Germany) solution for 30 min. The primary antibody was incubated overnight at 4 °C and detected with a fluorescence-labelled secondary antibody. The nucleus was stained with 2 μg/mL DAPI (#D9542, Sigma-Aldrich, Saint Louis, MI, United States). Imaging was performed using the CQ1 confocal imaging cytometer (Yokogawa) and analyzed using CellPathFinder software (Yokogawa). Employed antibodies are listed in Supplementary Table S2.

#### 2.12.2 3D immunofluorescence staining

3D immunofluorescence staining was performed following the manufacturer’s protocol for the MACS® Clearing Kit (Miltenyi Biotec, #130–126-719, Bergisch Gladbach, Germany), with minor adaptations. In brief, spheroids or PDAC tissue were fixed in 4% PFA for 1 h at room temperature and subsequently permeabilized with MACS® Permeabilization Solution for 6 h. Samples were incubated with primary antibodies for 48 h at 4 °C, followed by incubation with the respective secondary antibodies for 24 h at 4 °C. DAPI (2 μg/mL; Sigma-Aldrich, #D9542) was included during secondary antibody incubation for nuclear counterstaining. Dehydration was carried out through a graded ethanol series, with spheroids incubated for 3 h in each solution: 70% ethanol, 90% ethanol, and finally 100% ethanol. Following dehydration, samples were immersed in the MACS® Clearing Solution for 6 h to achieve optical transparency suitable for 3D imaging. For imaging, CQ1 Confocal Imaging Cytometer (Yokogawa) was used. 3D images were visualized using the 3D Viewer plugin in Fiji software (Schmid et al., 2010; Schindelin et al., 2012). Employed antibodies are listed in Supplementary Table S2.

### 2.13 Quantitative real-time PCR (qRT-PCR)

Total RNA was isolated utilizing the RNeasy MiniKit (#74104, Qiagen, Hilden, Germany) following the protocol provided by the manufacturer. RNA concentrations were quantified using the Tecan Spark NanoQuant plate (Tecan Group, Männedorf, Switzerland). Complementary DNA (cDNA) synthesis was performed with 1 µg of purified mRNA employing the QuantiTect Reverse Transcription Kit (#205311, Qiagen, Hilden, Germany). qRT-PCR was conducted using 50 ng of cDNA and the Luna® Universal qPCR Master Mix (#M3003L, New England BioLabs® Inc., Ipswich, MA, United States). Cycle threshold (Ct) values were determined using the ΔΔCT method, with sample normalization based on the housekeeping gene GAPDH (glyceraldehyde 3-phosphate dehydrogenase). Data analysis was carried out using the qPCRsoft 4.1 soft-ware (Analytic Jena, Jena, Germany). The primers utilized were procured from Eurofins Genomics (Ebersberg, Germany) and are listed in Supplementary Table S3.

### 2.14 Cell Viability Assay

#### 2.14.1 2D Cell Viability Assay

Cell viability was quantified using PrestoBlue HS Viability Reagent (#P50201, Thermo Fisher Scientific, Waltham, United States) according to the manufacturer’s instructions. Briefly, cells were seeded into 96-well plates (#83.3924, Sarstedt) and incubated under standard conditions (37 °C, 5% CO_2_, humidified atmosphere). At the end of treatment period, 10 µL of PrestoBlue reagent was added directly to each well containing 100 µL of culture medium and incubated for 60 min at 37 °C protected from light. Fluorescence was measured using Tecan spark microplate reader at 560 nm excitation and 590 nm emission. Synergy analysis of drug combinations was performed using Combenefit software (Cancer Research United Kingdom Cambridge Institute, Cambridge, United Kingdom).

#### 2.14.2 3D Cell Viability Assay

Cell viability was quantified using CellTiter-Glo® 3D Cell Viability Assay (#G9683, Promega, Madison, United States) according to the manufacturer’s instruction. Briefly, spheroids were transferred onto a white 96-well plate (#655083, Greiner Bio-one, Kremsmünster, Austria) with 50 µL medium. Equal volume of CellTiter-Glo 3D reagent was added directly to each well containing cell culture medium (1:1, v/v) and the contents were mixed on an orbital shaker for 5 min to ensure complete lysis and ATP release. For signal stabilization, plates were incubated for an additional 20 min at room temperature. Luminescence was recorded using TECAN spark micro plate reader.

### 2.15 Statistical analysis

All data analyses were performed using GraphPad Prism 8.0.2 software (GraphPad Software, Boston, MA, United States). Statistical comparisons between two groups were conducted using an unpaired t-test, whereas comparisons involving more than two groups were evaluated using one-way analysis of variance (ANOVA). Data are presented as mean ± standard error of the mean. A p-value ≤0.05 was considered statistically significant. Levels of significance are indicated as follows: p ≤ 0.05 (*), p ≤ 0.01 (**), p ≤ 0.001 (***), and p ≤ 0.0001 (****).

## 3 Results

**FIGURE 1:**
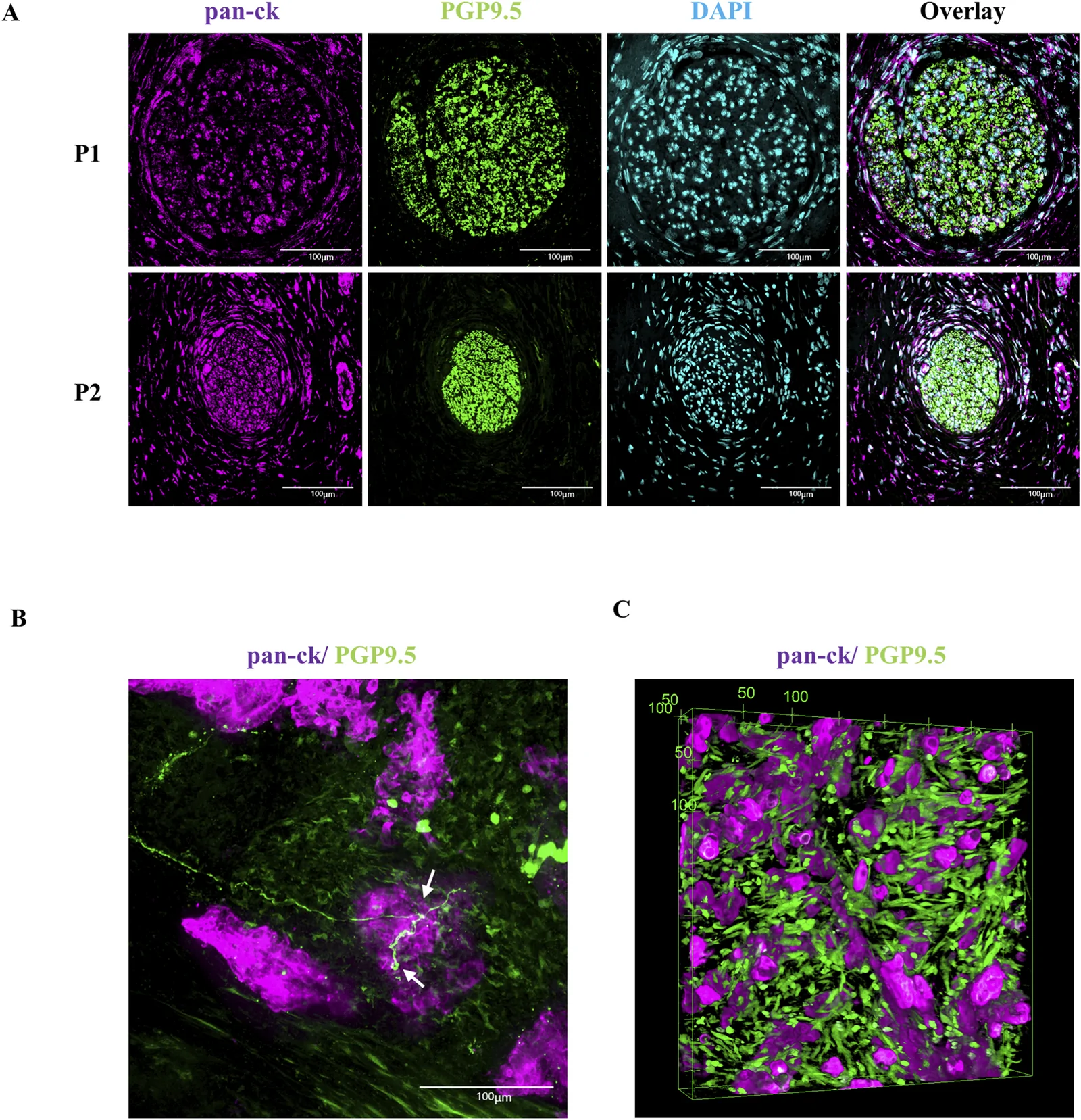
**(A)** Representative 2D immunofluorescence images illustrating the perineural invasion pattern of human PDAC tumor slices from two different PDAC patients. **(B,C)** 3D visualization of human PDAC explants acquired as z-stacks with 1 µm interval steps, shown as maximum intensity projection (MIP) **(B)**, or individual z-stack slice reconstruction generated with ImageJ **(C)**. Cancer cells were labelled with pan-ck (purple), neural structures with PGP9.5 (green) and nuclei are counterstained with DAPI (blue). White arrows highlight axonal structure infiltrating the tumor lesion. Scale bar is 100 µm.

2D immunofluorescence (IF) staining of human PDAC tissue sections, with neuronal structures labeled by PGP9.5 (green) and tumor cells by pan-cytokeratin (pan-CK; purple), revealed that tumor lesions were in close proximity to neuronal structures, consistent with the characteristic pattern previously described for perineural invasion (PNI) (Lovecek et al., 2025). Furthermore, individual tumor cells were detected within the nerve structures (Figure 1A). These findings were confirmed in five different PDAC samples (n = 5) (Supplementary Figure S1). To further investigate the spatial organization of neural structures in PDAC, we performed 3D IF imaging of human PDAC explants derived from tumor resection. In contrast to formalin-fixed PDAC slices, which are limited to 2D analysis and may introduce fixation-induced artifacts, imaging of fresh explants enables the preservation of the native 3D architecture of the tumor. Optical tissue clearing facilitated visualization throughout the entire tumor specimen. PDAC explants were imaged at 1 µm intervals to generate high-resolution z-stacks, allowing for precise spatial mapping of cancer cells (pan-ck, purple) and neural structures (PGP9.5, green). These are presented as a maximum intensity projection (MIP) (Figure 1B), and as individual optical sections, reconstructed in 3D (Figure 1C). 3D reconstruction revealed that tumor lesions form an interconnected network. Moreover, tumor lesions are extensively traversed by neural structures, providing unprecedented 3D insight into the spatial organization of neural infiltration in human PDAC tissue.

**FIGURE 2:**
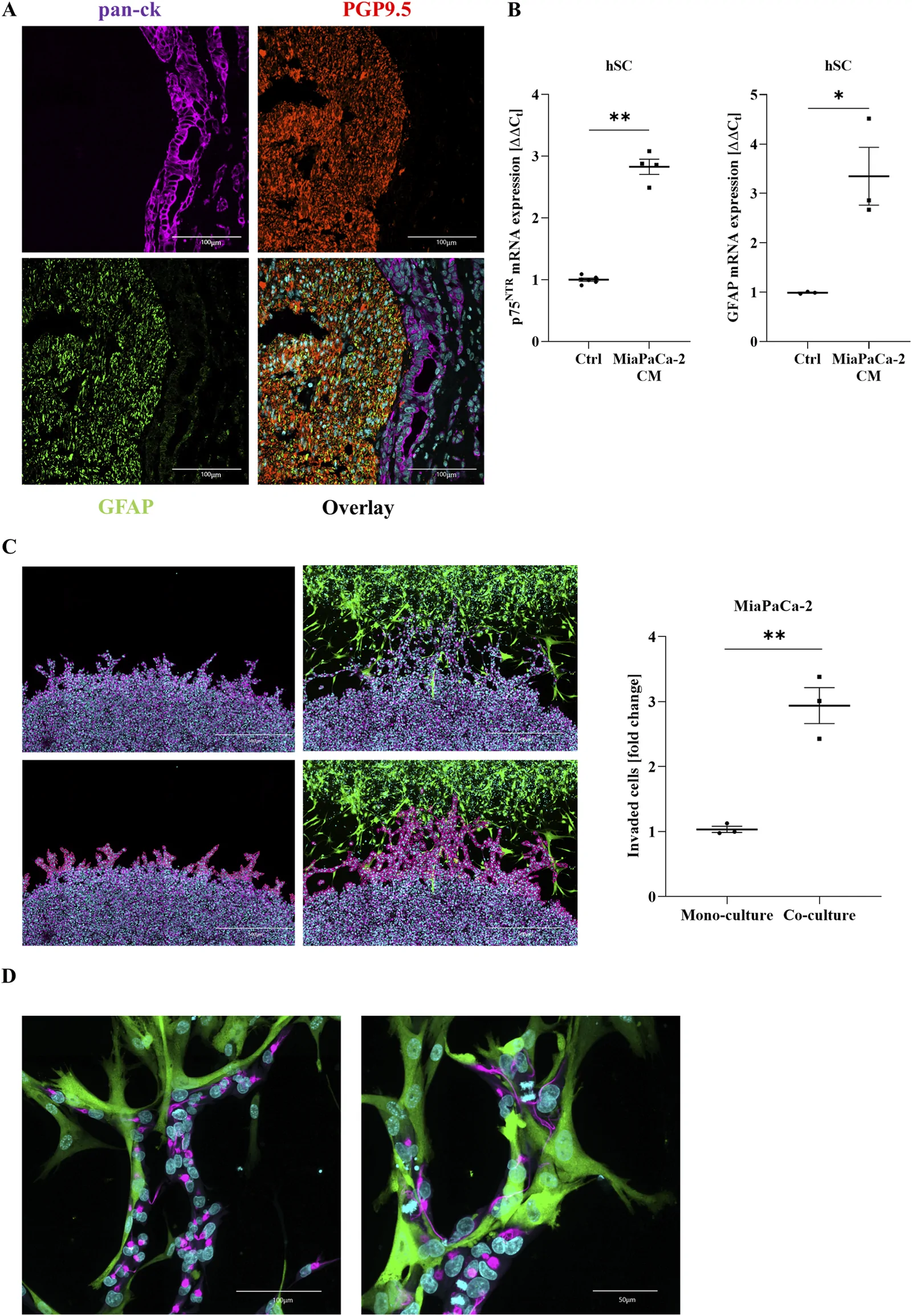
**(A)** Representative immunofluorescence staining of human PDAC slices with Schwann cells labeled with GFAP (green) within neural structures marked by PGP9.5 (red). Cancer cells are labeled with pan-ck (purple), and nuclei are counterstained with DAPI (blue). Scale bar is 100 µm. **(B)** Primary human Schwann cells (hSC) were treated with MiaPaCa-2 CM for 48 h mRNA expression of GFAP and p75NTR was quantified using the ΔΔCt method. **(C)** Spherical invasion assay was performed with MiaPaCa-2 cells and hSC and monitored for 72 h. MiaPaCa-2 cells are labelled with pan-ck (purple), Schwann cells with PGP9.5 (green), and nuclei are counterstained with DAPI (blue). Quantification of invaded MiaPaCa-2 cells was assessed using the CellPathFinder software. Scale bar is 500 µm. **(D)** High-magnification images of MiaPaCa-2 cells and Schwann cell track formation. Scale bar left is 100 μm, right is 50 µm. Error bars indicate the standard errors of the mean of at least three independent experiments, with * = p ≤ 0.05; ** = p ≤ 0.01; *** = p ≤ 0.005; **** = p ≤ 0.0001.

To characterize the cellular subtypes of neural structures within human PDAC tissue slices, we first focused on Schwann cells. Schwann cells (hSC), labeled with GFAP, were identified within PGP9.5-positive neural structures in human PDAC tissue slices (Figure 2A). *In vitro* mRNA expression analysis of primary human Schwann cells (hSC) revealed a significant upregulation of p75^NTR^ (2.9-fold) and GFAP (3.1-fold) after 48 h of treatment with MiaPaCa-2 conditioned medium (CM), confirming a phenotypic shift towards a tumor-associated GFAP^+^/p75NTR^high^ Schwann cell state (Figure 2B). To assess whether Schwann cells directly influence tumor cell invasion, we established a co-culture set-up consisting of hSC and MiaPaCa-2 cells. Under these conditions, MiaPaCa-2 cells exhibited a 3-fold increase in the number of invading cells (Figure 2C). High-resolution imaging revealed that invading MiaPaCa-2 cells established direct physical contact with hSC and preferentially migrated along hSC, utilizing them as guiding tracks for directional invasion (Figure 2D).

**FIGURE 3:**
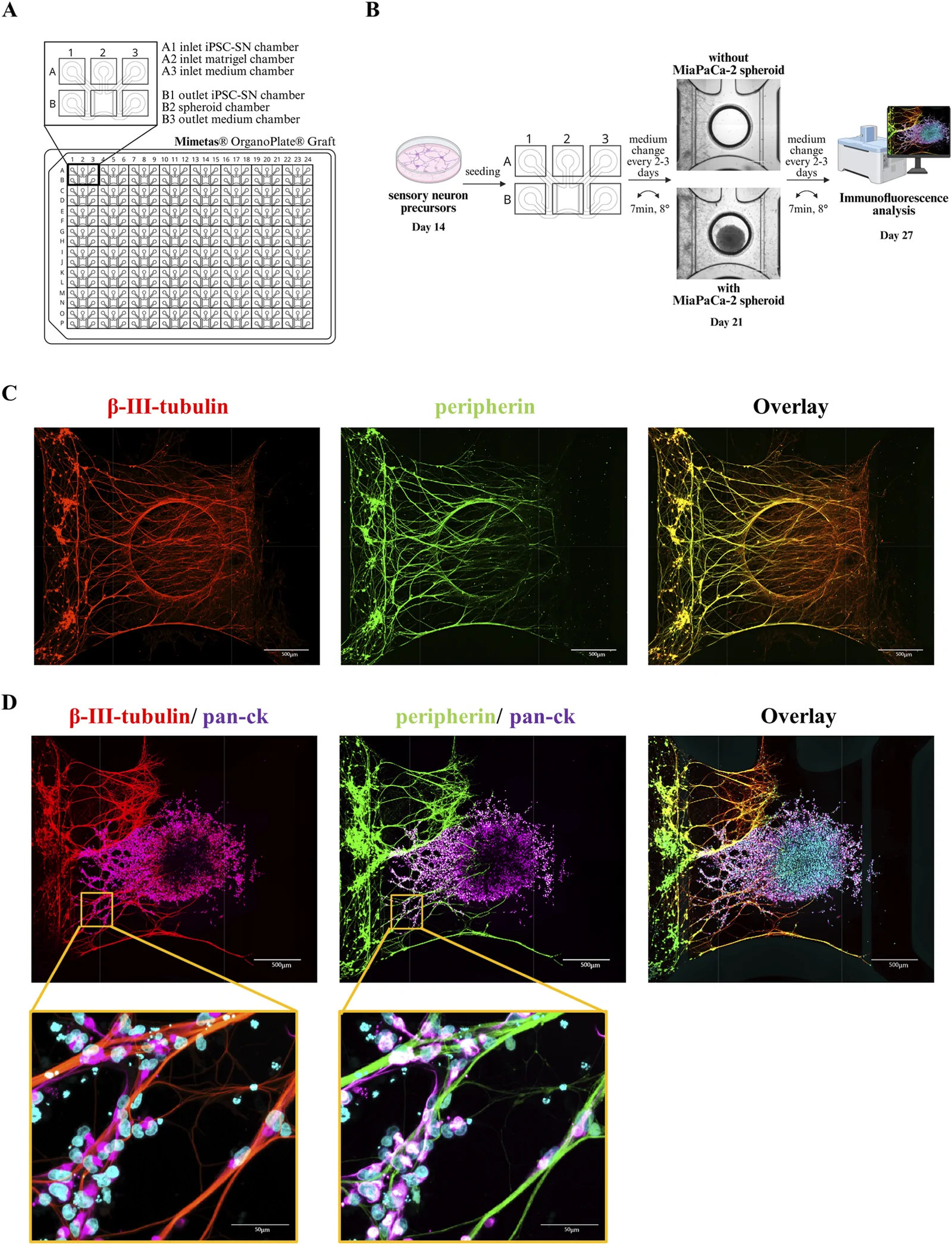
**(A)** Schematic illustration of OrganoPlate® Graft chamber layout. **(B)** Schematic representation of the co-culture workflow of iPSC-derived sensory neurons (SN) and MiaPaCa-2 spheroids on the OrganoPlate® Graft. **(C)** Representative immunofluorescence staining of iPSC-derived SN matured on the OrganoPlate®. Axons are labelled with β-III-tubulin (red) and peripherin (green). Scale bar is 500 µm. **(D)** Co-culture of iPSC-derived SN and MiaPaCa-2 spheroids after 7 days. Axons are labeled with peripherin (green) and β-III-tubulin (red), MiaPaCa-2 cells with pan-ck (purple), and nuclei are counterstained with DAPI (blue). Scale bar is 500 µm. Lower panels show high-magnification images. Scale bar is 100 µm. Schematic illustrations are created with BioRender.

Expanding our focus beyond Schwann cells, we hypothesized that sensory neurons (SN) directly guide cancer cell invasion via axon-guidance mechanisms. To test this, we developed a co-culture system comprising of iPSC-derived human SN and MiaPaCa-2 spheroids using the OrganoPlate® Graft within the MIMETAS® microfluidic system. The differentiation and functional characterization of iPSC-derived SN was confirmed by the expression of mature sensory neuron markers peripherin and β-III-tubulin (Supplementary Figure S2). As shown in Figure 3A, each OrganoPlate® Graft contains 40 independent microfluidic chips integrated into a standard 384-well plate format, enabling parallelized culture under controlled perfusion conditions. This set-up facilitates the spatially defined co-culture of iPSC-derived SN and MiaPaCa-2 spheroids within compartmentalized chambers (Figure 3A). Figure 3B illustrates the experimental workflow. For the co-culture of iPSC-derived SN and MiaPaCa-2 spheroids, the spheroids were precisely integrated into the spheroid chambers containing day-21 iPSC-derived SN (Figure 3B). The iPSC-derived SN extended extensive neurite networks throughout the microchannels, forming dense and highly organized neuronal architectures. The acquisition of mature neuronal characteristics was confirmed by immunostaining for peripherin (green) and β-III-tubulin (red) (Figure 3C). After 7 days of co-culture, MiaPaCa-2 cells exhibited pronounced axon-guided invasion along the iPSC-derived SN networks (Figure 3D).

**FIGURE 4:**
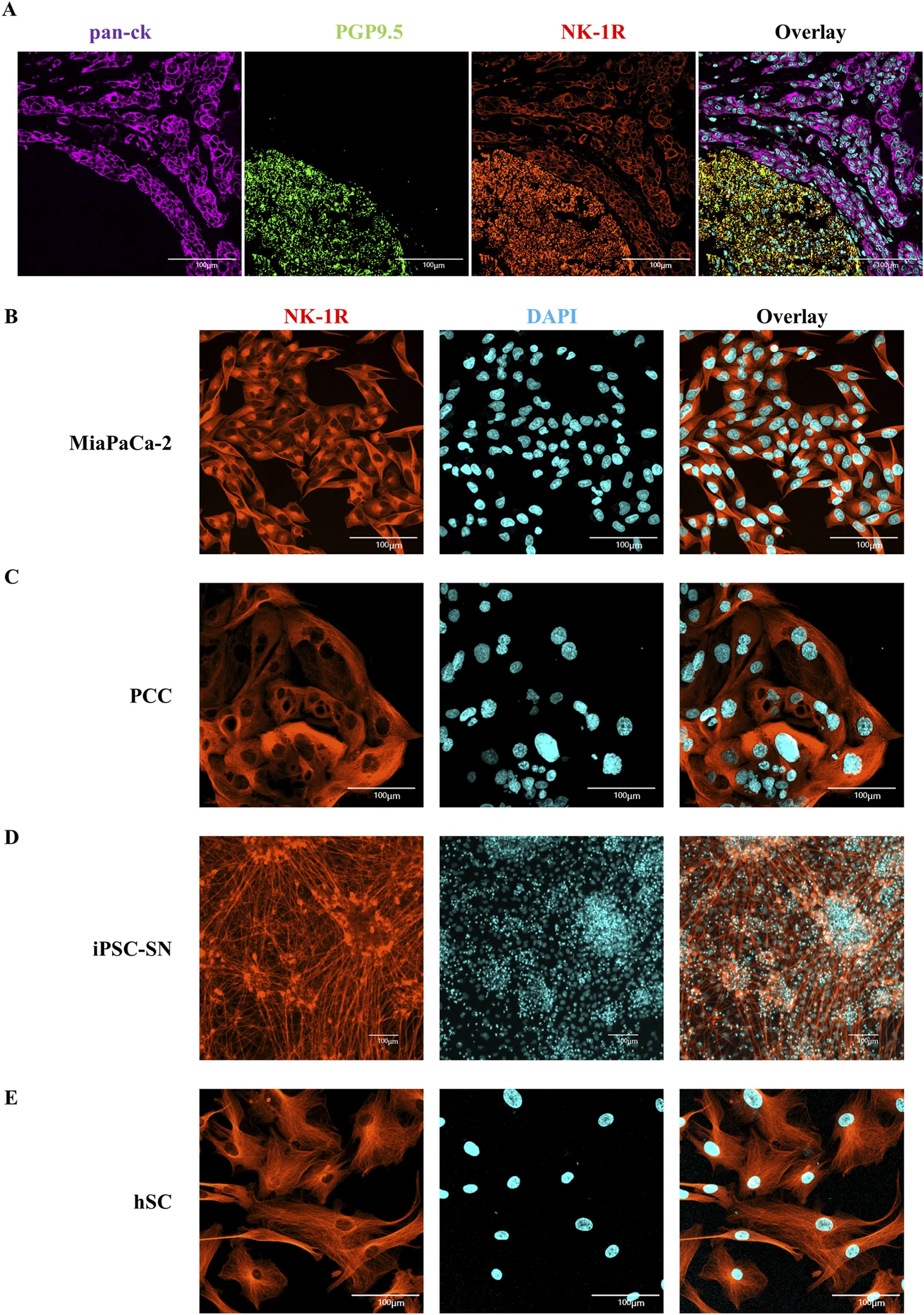
Representative immunofluorescence images of human PDAC slices **(A)**, MiaPaCa-2 cells **(B)**, primary pancreatic cancer cells (PCC) **(C)**, iPSC-derived sensory neurons (SN) **(D)** and Schwann cells (hSC) **(E)**. Cancer cells are labelled with pan-ck (purple), neural structures with PGP9.5 (green). NK-1R is shown in red. Nuclei are counterstained with DAPI (blue). Scale bar is 100 µm.

Previous studies have shown that NK-1R is overexpressed in PDAC tissue (Li et al., 2013a; Friess et al., 2003a). To corroborate these findings, NK-1R expression was confirmed via immunofluorescence in human PDAC tissue slices. NK-1R was robustly expressed by tumor cells (pan-ck, purple) as well as neural structures (PGP9.5, green) (Figure 4A). To additionally confirm constitutive NK-1R expression in pancreatic carcinoma cells, we analyzed the PDAC cell line MiaPaCa-2 cells (Figure 4B) and primary tumor cells directly obtained from primary resected tissue (Figure 4C) Immunofluorescence staining revealed NK-1R expression in both tumor cell models, validating MiaPaCa-2 cells as suitable for *in vitro* modelling of NK-1R–mediated tumor–nerve interactions. Furthermore, we demonstrated that NK-1R is highly expressed by our iPSC-derived SN (Figure 4D). Notably, we also detected strong NK-1R expression in hSC, leading us to hypothesize that NK-1R may additionally contribute to Schwann cell-mediated invasion (Figure 4E).

**FIGURE 5:**
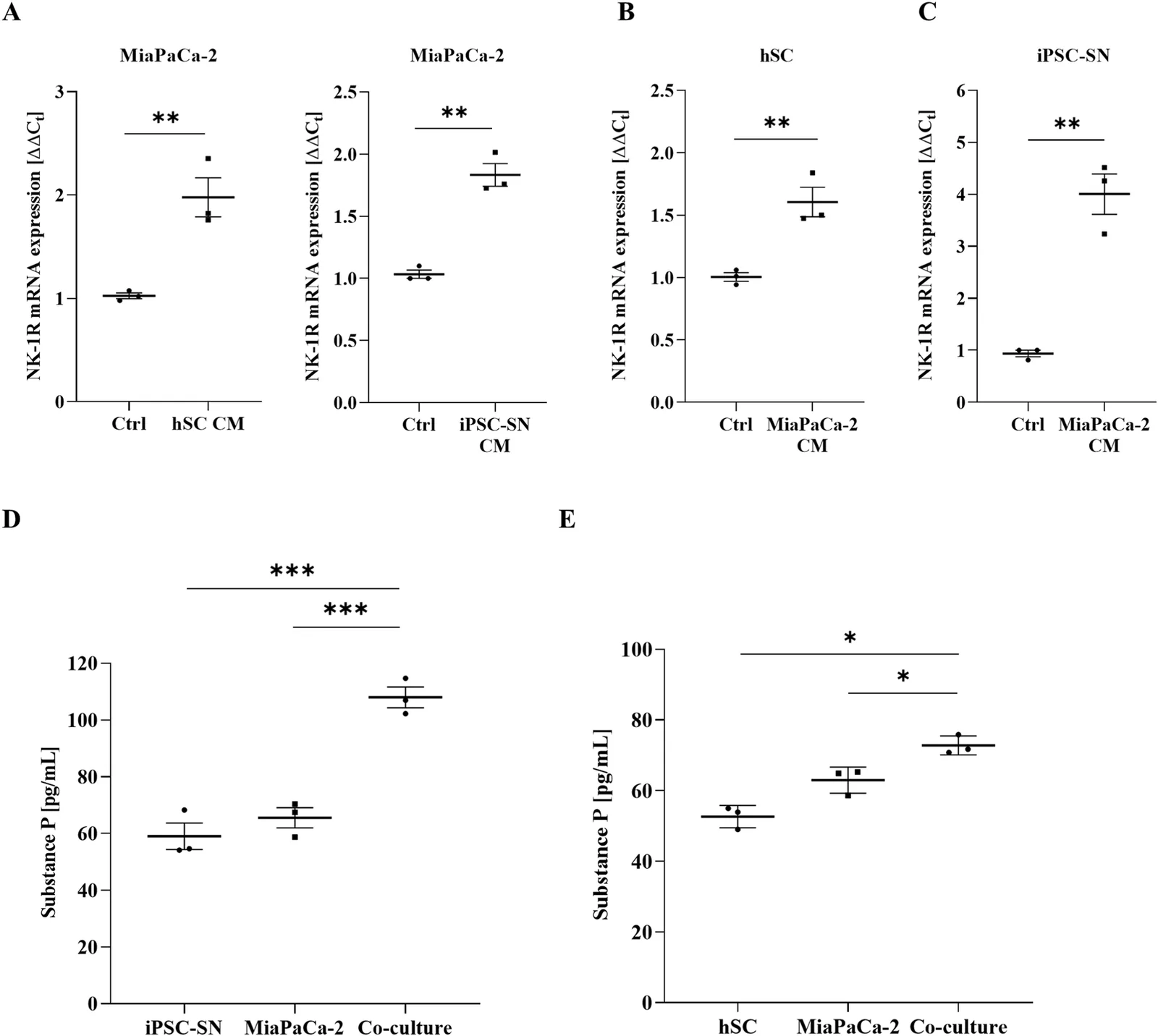
**(A)** MiaPaCa-2 cells were treated with CM from Schwann cells (hSC) and iPSC-derived sensory neurons (SN) for 48 h. **(B)** hSC were treated for 48 h and **(C)** iPSC-derived SN for 24 h with MiaPaCa-2 CM. mRNA expression of NK-1R was quantified using the ΔΔCt method. **(D,E)** Supernatant of mono or co-culture was collected after 24 h and SP concentration was quantified via ELISA. Error bars indicate the standard errors of the mean of at least three independent experiments, with * = p ≤ 0.05; ** = p ≤ 0.01; *** = p ≤ 0.005; **** = p ≤ 0.0001.

To assess whether sensory neurons or Schwann cells regulate NK-1R expression in tumor cells, we performed mRNA expression analysis in MiaPaCa-2 cells following exposure to CM derived from iPSC-derived SN or hSC. Exposure to CM from either neural cell type resulted in a significant upregulation of NK-1R mRNA expression in MiaPaCa-2 cells (2-fold for hSC CM, and 1.8-fold for iPSC-derived SN CM) (Figure 5A). Next, we next analyzed NK-1R mRNA expression in hSC and iPSC-derived SN following exposure to CM derived from MiaPaCa-2 cells. Notably, MiaPaCa-2 CM significantly upregulated NK-1R mRNA in hSC (1.6-fold) and iPSC-derived SN (4-fold) (Figures 5B,C). Given the bidirectional regulation of NK-1R mRNA expression between tumor and neural cells, we next investigated whether this was accompanied by changes in SP secretion. SP protein levels in the supernatants of both co-cultures (MiaPaCa-2/iPSC-derived SN and MiaPaCa-2/hSC) were significantly increased compared to their respective mono-culture conditions (Figures 5D,E).

**FIGURE 6:**
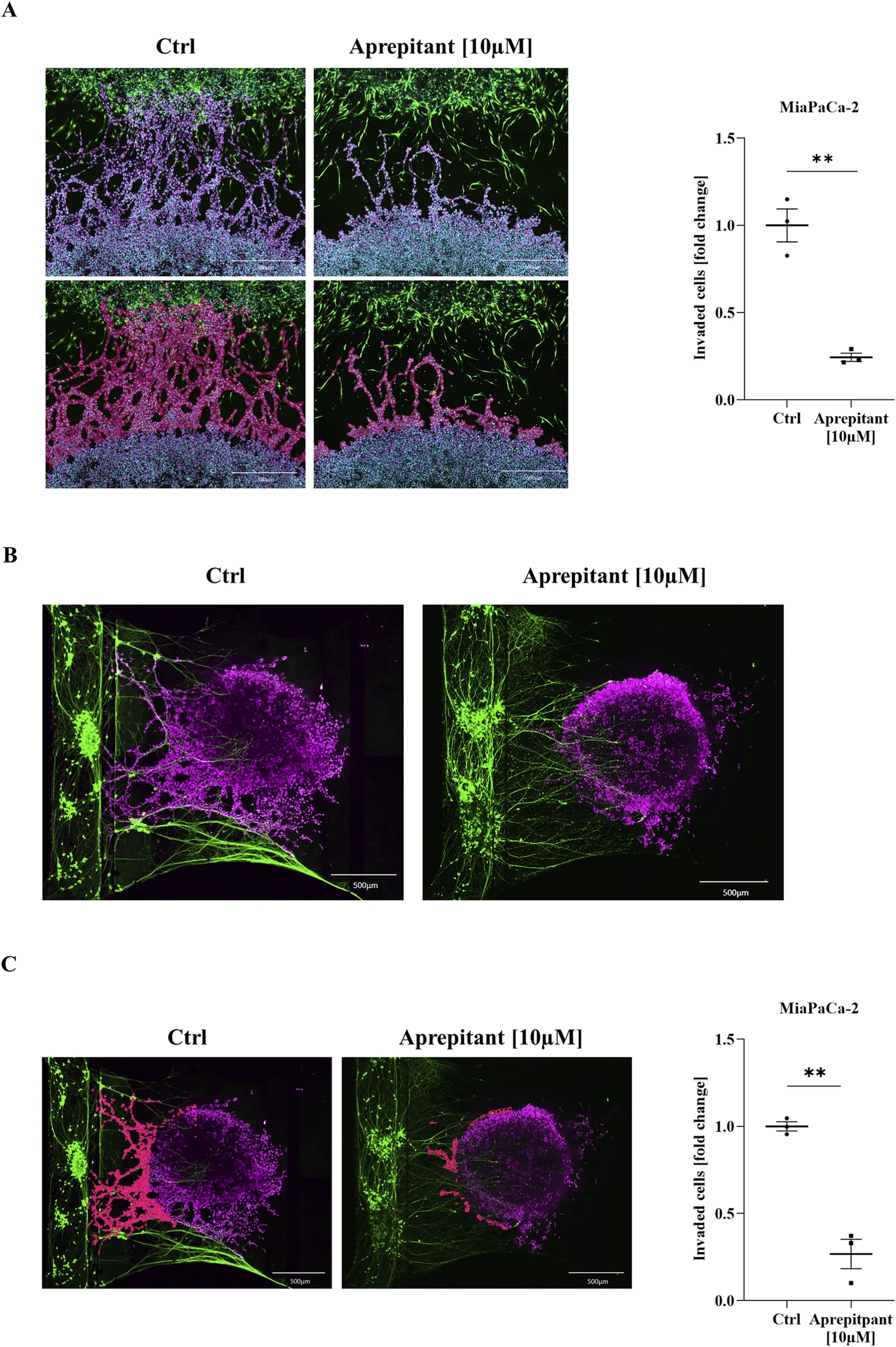
**(A)** Spherical invasion assay of MiaPaCa-2 cells co-cultured with Schwann cells (hSC) and treated with or without 10 µM aprepitant. Cultures were monitored over 72 h. MiaPaCa-2 cells are labeled with pan-ck (purple), hSC with PGP9.5 (green), and nuclei are counterstained with DAPI (blue). **(B)** Co-culture of iPSC-derived sensory neurons (SN) and MiaPaCa-2 spheroid on the OrganoPlate® Graft for 1 week with or without 10 µM aprepitant. iPSC-derived SN are labeled with PGP9.5 (green), MiaPaCa-2 cells with pan-ck (purple), and nuclei with DAPI (blue). **(C)** Quantification of axon-guided MiaPaCa-2 cells shown in **(B)**. Error bars indicate the standard errors of the mean of at least three independent experiments, with * = p ≤ 0.05; ** = p ≤ 0.01; *** = p ≤ 0.005; **** = p ≤ 0.0001. Scale bar is 500 µm.

To evaluate the therapeutic relevance of NK-1R, we utilized aprepitant, a clinically approved NK-1R antagonist, in our co-culture invasion assays. Aprepitant was applied at a subtoxic concentration (Supplementary Figure S3). Treatment with aprepitant led to a marked reduction in the number of invading MiaPaCa-2 cells in the Schwann cell co-cultures (0.23-fold) (Figure 6A). Similarly, aprepitant significantly impaired the axon-guided invasion of MiaPaCa-2 cells in the iPSC-derived SN/MiaPaCa-2 spheroid co-cultures (0.25-fold) (Figures 6B,C). Treatment of MiaPaCa-2 cells with recombinant substance P induced a modest but significant increase in mRNA vimentin levels. This effect was abolished by co-treatment with aprepitant (Supplementary Figure S4).

**FIGURE 7:**
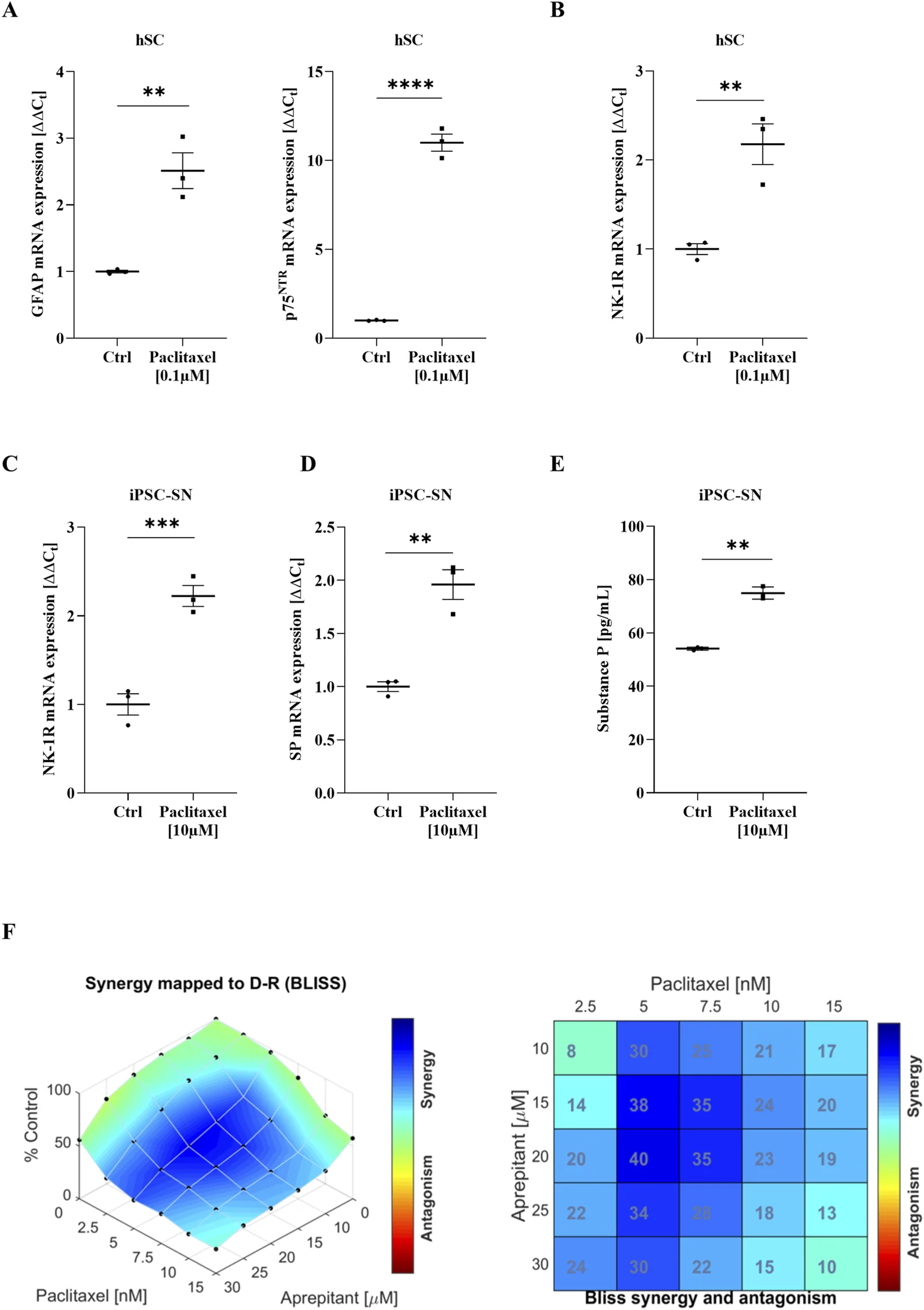
**(A,B)** Schwann cells (hSC) were treated with 0.1 µM paclitaxel for 48 h and GFAP, p75NTR and NK-1R mRNA levels were quantified using the ΔΔCt method. **(C)** iPSC-derived sensory neurons (SN) were treated with 10 µM paclitaxel for 48 h and NK-1R mRNA expression were quantified using the ΔΔCt method. **(D)** iPSC-derived SN were treated with 10 µM paclitaxel for 2 *h. SP* mRNA levels were quantified using the ΔΔCt method. **(E)** iPSC-derived SN were treated with 10 µM paclitaxel for 2 h. Supernatant was collected and SP concentrations were quantified via ELISA. **(F)** MiaPaCa-2 cells were treated for 72 h with combinations of paclitaxel and aprepitant. Synergistic effects were analyzed using Combenefit software using BLISS analysis. Error bars indicate the standard errors of the mean of at least three independent experiments, with * = p ≤ 0.05; ** = p ≤ 0.01; *** = p ≤ 0.005; **** = p ≤ 0.0001.

Paclitaxel, a first-line chemotherapeutic agent for PDAC patients, is known to cause chemotherapy-induced neuropathic pain. This condition is associated with Schwann cell dedifferentiation, characterized by the upregulation of GFAP and p75NTR (Deborde et al., 2022). mRNA expression analysis revealed that paclitaxel significantly increased the expression of p75NTR (2.6-fold) and GFAP (10.3-fold) in hSC (Figure 7A). Next, we examined whether paclitaxel modulates the SP/NK-1R signaling axis within neural cells. Treatment with paclitaxel resulted in the significant upregulation of NK-1R mRNA expression in both hSC (2.1-fold) and iPSC-derived SN (2.2-fold) (Figures 7B,C). Additionally, paclitaxel significantly upregulated mRNA expression of substance P (SP) in iPSC-derived SN (1.9-fold) (Figure 7D). Consistent with this, ELISA analysis of iPSC-derived SN supernatants showed a significant increase in SP secretion following paclitaxel treatment (Figure 7E). Together, these findings demonstrate that paclitaxel not only enhances NK-1R expression but also stimulates SP release from sensory neurons. Given that NK-1R has been implicated in chemoresistance (García-Aranda et al., 2022), we next investigated whether pharmacological inhibition with aprepitant modulates cytotoxicity and enhances the response to paclitaxel in MiaPaCa-2 cells. Treatment of MiaPaCa-2 cells with aprepitant alone reduced cell viability in a dose-dependent manner, with an IC_50_ value of 26 µM after 72 h (Supplementary Figure S5). Furthermore, a combinatorial dose–matrix approach revealed interaction effects between paclitaxel and aprepitant across all tested concentrations (Figure 7F). As shown in the Bliss synergy analysis (Figure 7F), the strongest synergistic effect was identified at 5 nM paclitaxel and 20 µM aprepitant.

## 4 Discussion

Perineural invasion (PNI) represents a defining pathological feature of PDAC associated with poor prognosis and is increasingly recognized as a consequence of active and reciprocal signaling between tumor cells and neural components within the tumor microenvironment (Schouten et al., 2025; Meng et al., 2026; Chen et al., 2019; Cha et al., 2023). In this context, both sensory neurons and Schwann cells have emerged as critical regulators that shape a pro-invasive tumor-neural niche, thereby facilitating PNI (Zhang Y. et al., 2025; Ebrahim et al., 2025). However, current mechanistic insights are largely derived from genetically engineered mouse models, particularly the KPC model, which frequently fail to faithfully recapitulate key features of human PNI and exhibit limited translational relevance (Wang et al., 2023; Demir et al., 2015). As a result, the cellular and molecular mechanisms underlying PNI in human PDAC remain incompletely understood.

In line with previous studies, we demonstrate that Schwann cells are an integral component of intratumoral neural structures (Ebrahim et al., 2025). Moreover, Deborde et al. previously identified Schwann cell–mediated guidance of directed tumor cell migration (Deborde et al., 2022). In our study, we corroborate these findings by demonstrating enhanced Schwann cell–mediated tumor cell invasion and confirming that Schwann cells act as guiding tracks in co-cultures composed of primary human Schwann cells (hSC) and MiaPaCa-2 cells. Although Thiel et al. recently provided the first transcriptomic dataset of intratumoral neurons in a orthotopic xenograft mouse model, to date, comparable human functional and transcriptomic data remain currently unavailable (Thiel et al., 2025). To address this gap, we established, a human 3D microfluidic model of PNI by co-culturing iPSC-derived SN with MiaPaCa-2 spheroids. Using this system, we demonstrate that PDAC cells undergo axon-guided migration along human sensory neurons, resulting in enhanced tumor cell dissemination.

The SP/NK-1R axis is well-established in the pathophysiology of neuropathic pain, including cancer-associated pain (Esteban et al., 2006). Previous studies have been shown that SP/NK-1R signaling promotes tumor proliferation and migration, and is negatively associated with survival in multiple solid malignancies (Al-Keilani et al., 2023; Zhang et al., 2023; Chen et al., 2016). In PDAC, elevated expression of NK-1R correlates with disease progression (Friess et al., 2003b). Our data extend these findings by demonstrating robust NK-1R expression in human PDAC slices, and in pancreatic cancer cells, indicating that PDAC cells are highly responsive to neuropeptidergic substance P (SP) signaling. Importantly, we observed *in vitro* that iPSC-derived sensory neurons and primary human Schwann cells significantly increased NK-1R expression in tumor cells, suggesting that neural structures actively prime tumor cells to respond to SP. In addition to its effects on tumor cells, our study demonstrates that NK-1R signaling plays a role in Schwann cell dedifferentiation. While SP/NK-1R signaling has been extensively studied in astrocytes and microglia of the central nervous system, its role in Schwann cells has remained completely unexplored (Burmeister et al., 2017). We demonstrated that Schwann cells exhibit strong NK-1R expression, which was upregulated via exposure to MiaPaCa-2 conditioned medium (CM). Furthermore, in co-cultures composed of MiPaCa-2/sensory neurons and MiaPaCa-2/Schwann cells, supernatants showed increased SP protein levels compared to their respective mono-culture conditions. Taken together, these findings suggest that NK-1R/SP signaling represent a key mechanism of PNI in human PDAC. Future studies may benefit from the use of a tri-culture model comprising tumor cells, Schwann cells, and sensory neurons, which may more closely recapitulate the complexity of the human tumor–neural microenvironment.

To assess the functional role of NK-1R in cancer cells, we aim to investigated how its pharmacological antagonism influences tumor cell invasion within our co-culture models. While pharmacological inhibition of NK-1R with the FDA approved antagonist aprepitant is well recognized for its anti-tumor effects, including the induction of apoptosis and inhibition of cancer progression, its role in regulating tumor cell invasion has been less extensively explored (Muñoz and Rosso, 2010). In non-PDAC malignancies such as glioblastoma and osteosarcoma, NK-1R antagonism by aprepitant has been shown to impair cancer cell migration (Engür-Öztürk et al., 2025; Alsaeed et al., 2022). We demonstrated that NK-1R inhibition via aprepitant significantly reduces MiaPaCa-2 cell invasion in both co-culture systems composed of MiaPaCa-2/sensory neurons and MiaPaCa-2/Schwann cells. Our findings provide the observation that NK-1R blockade effectively impairs both Schwann cell–mediated tumor invasion and the axon guidance of tumor cells, thereby underscoring the therapeutic potential of repurposing aprepitant to improve PDAC patient outcomes.

Given the established role of the NK-1R axis in neuropathic pain, and evidence that paclitaxel, a first-line chemotherapeutic agent for PDAC, induces chemotherapy-associated neuropathic pain through SP upregulation in preclinical models (Giri et al., 2024), we investigated whether paclitaxel modulates the NK-1R/SP signaling axis. Specifically, we examined its effects on Schwann cell dedifferentiation and the NK-1R/SP axis in Schwann cells and sensory neurons. In line with previous reports, we demonstrate that paclitaxel induces Schwann cell dedifferentiation, as evidenced by the upregulation of GFAP and p75NTR mRNA expression (Schinke et al., 2026; Imai et al., 2017). Furthermore, we show that paclitaxel upregulates NK-1R mRNA expression in both Schwann cells and sensory neurons. Moreover, paclitaxel increased SP secretion from sensory neurons, demonstrating that it modulates the SP/NK-1R axis in neuronal cells. Previous studies have suggested that NK-1R signaling contributes to chemoresistance; however, combination-based studies involving NK-1R antagonism via aprepitant remain limited (García-Aranda et al., 2022). Given its well-established clinical safety profile, aprepitant represents a highly attractive candidate for drug repurposing (Muñoz and Coveñas, 2020; Robinson et al., 2023). To address this, we investigated the combinatorial effects of aprepitant and paclitaxel and demonstrate, that aprepitant synergistically enhances the cytotoxic effects of paclitaxel in MiaPaCa-2 cells. These findings suggest that combining aprepitant with paclitaxel may improve therapeutic efficacy in PDAC patients, particularly during the initial phase of treatment, when paclitaxel-induced molecular effects are most prominent (da Costa et al., 2020).

Bridging the gap between preclinical mouse models and human translational systems remains a critical challenge in biomedical research. The development of human-relevant model systems is therefore essential to improve translational predictability, a gap that we address in the present study. Here, we establish a fully human-based functional model system comprising sensory neurons and tumor cells that recapitulates key features of PNI in PDAC, thereby representing a novel New Approach Methodology (NAM) for the study of tumor–nerve interactions. NAMs not only strengthen the ethical framework of preclinical research but also offer enhanced translational fidelity, significantly improving predictive power, mechanistic resolution, and clinical relevance (Edwards et al., 2025). Our work is fully aligned with the principles of Replacement, Reduction, and Refinement (3Rs).

Nevertheless, several limitations of this study should be acknowledged. First, our experiments were primarily conducted *in vitro* and therefore do not fully recapitulate the complexity of the *in vivo* tumor microenvironment, including the contributions of immune cell populations, vascular components, and systemic factors that are known to critically shape the tumor microenvironment (Katt et al., 2016). In addition, the use of established PDAC cell lines, while enabling controlled mechanistic studies, does not fully capture the profound tumoral heterogeneity characteristic of pancreatic cancer. Future studies employing patient-derived organoids, complemented by larger and more diverse patient cohorts, will be essential to validate and extend these findings in a more physiologically and clinically relevant context. Moreover, while our data implicate NK-1R signaling as a regulator of tumor invasiveness, the precise downstream mechanisms remain incompletely defined. Additionally, it has been reported that full-length and truncated NK-1R isoforms may exhibit distinct functional properties; however, our study did not differentiate between receptor variants, as our focus was on the overall functional role of NK-1R signaling within the tumor-neural niche (Beirith et al., 2021a). Previous studies have demonstrated that NK-1R signaling activates oncogenic pathways, including MAPK/ERK, PI3K/Akt, and NF-κB signaling cascades, leading to tumor cell survival, invasion and chemotherapy resistance (Huang et al., 2018; Li et al., 2013a; Beirith et al., 2021a; Engür-Öztürk et al., 2025; Ji et al., 2022b; Ghahremanloo et al., 2021). ERK1/2 and Akt phosphorylation have been linked to increased motility and metastatic potential (Huang et al., 2018), partly through the induction of MMP-2 and subsequent extracellular matrix remodeling (Li et al., 2013a). Furthermore, in pancreatic cancer cells, substance P-mediated invasion has also been linked to increased Erk1/2 signaling associated with epithelial–mesenchymal transition (EMT) by upregulation of vimentin and Twist (Huang et al., 2018). Linking NK-1R signaling to EMT-associated processes, we observed a modest but significant increase in vimentin mRNA levels following substance P treatment. Importantly, this effect was reversed by NK-1R blockade with aprepitant, further supporting the involvement of the SP/NK-1R axis in regulating EMT-related molecular changes. However, further elucidation of downstream signaling pathways activated by NK-1R in pancreatic cancer, particularly through transcriptomic analyses in patient-derived models, will be essential to clarify its role in tumor–nerve interactions and disease progression. Importantly, delineating these mechanisms will not only deepen our understanding of NK-1R biology in the context of tumor–nerve crosstalk but may also uncover actionable nodes for novel therapeutic intervention in the future.

Collectively, our findings confirm and extend previous work by implicating the SP/NK-1R axis as one potential driver of PNI in PDAC in a human *in vitro* set up. By leveraging a human 3D microfluidic co-culture PNI model, we provide additional evidence that NK-1R signaling contributes to sustaining bidirectional tumor-neural crosstalk. Pharmacological inhibition of NK-1R not only suppressed PNI but also synergistically enhanced paclitaxel-induced cytotoxicity, highlighting a dual vulnerability in both neural invasion and chemoresistance. Our data demonstrate that therapeutic targeting of NK-1R may therefore confer multiple benefits by overcoming chemoresistance and suppressing PNI while simultaneously alleviating cancer-associated pain, thereby providing a strong translational rationale for combining NK-1R antagonists with paclitaxel in PDAC. Beyond these immediate findings, the described human-relevant model systems establish a powerful platform for transcriptomic profiling, paving the way for the discovery of novel, clinically actionable targets involved in PDAC-associated perineural invasion.

## Data Availability

The original contributions presented in the study are included in the article/Supplementary Material, further inquiries can be directed to the corresponding author.
