# Supplementary material for: The substance P/ Neurokinin-1 receptor signaling drives perineural invasion in pancreatic ductal adenocarcinoma (PDAC)
Source: Front Cell Dev Biol. 2026 Aug 19;14:1876023. doi: 10.3389/fcell.2026.1876023 (PMC13534051; doi:10.3389/fcell.2026.1876023)
Supplement: Supplementary file 1 [file DataSheet1.docx]

Supplementary Material

# 1 Supplementary Data

## 1.1 Supplementary Tables

Table 1 Clinicopathological characteristics of PDAC patient cohort used for fresh tissue analyses.

| Patient | Grading | UICC stage | Neoadjuvant therapy | Surgical resection | Perineural invasion |
| --- | --- | --- | --- | --- | --- |
| 1 | G3 high grade | IV | No | Yes | Yes |
| 2 | G3 high grade | IB | No | Yes | Yes |
| 3 | G2 low grade | IIB | No | Yes | Yes |

Table 2 List of employed antibodies, catalogue numbers and vendors.

| Primary Antibody | Host | Catalogue number | Vendor |
| --- | --- | --- | --- |
| GFAP | rabbit | #ab68428 | Abcam |
| Pan-Cytokeratin-VioR667 | recombinant | #130-133-439 | Miltenyi |
| Peripherin | rabbit | #ab246502 | Abcam |
| PGP9.5 | rabbit | #AB108986 | Abcam |
| NK-1R | mouse | #39-6100 | Thermo Scientific |
| β-III-Tubulin | mouse | #ab78078 | Abcam |
| **Secondary Antibody** | **Host** | **Catalogue number** | **Vendor** |
| Alexa Flour 488 (H+L) anti-rabbit | donkey | #A-21206 | Invitrogen |
| Alexa Flour 546 (H+L) anti-rabbit | donkey | #A-10040 | Invitrogen |
| Alexa Flour 647 (H+L) anti-mouse | donkey | #A-31571 | Invitrogen |

Table 3 A detailed summary of the oligonucleotides utilized, including target gene and corresponding primer sequences.

| Target gene | Forward (5´-3´) | Reverse (5´-3´) |
| --- | --- | --- |
| GAPDH | TGCACCACCAACTGCTTAGC | GGCATGGACTGTGGTCATGAG |
| GFAP | AGGTCCATGTGGAGCTTGAC | GCCATTGCCTCATACTGCGT |
| Nav1.7 | GTGGAAGGATTGTCAGTTCTGCG | GCCAACACTAAGGTGAGGTTACC |
| Nav1.8 | TGGCAGATGACCTGGAAGAACC | CGATACGGTAGCAAGTCTTGCG |
| NK-1R | GCCTGTTCTACTGCAAGTTCCAC | CACAGATGACCACTTTGGTGGC |
| P75NTR | CCTCATCCCTGTCTATTGCTCC | GTTGGCTCCTTGCTTGTTCTGC |
| SP | TTACTGGTCCGACTGGTACGAC | CAAAGAACTGCTGAGGCTTGGG |
| TRPM8 | CTGGTTGCGAACTTCCGAAGAG | GGTGCCGAGTAATAGGAGACAC |
| TRPV1 | GTGGACAGCTACAGTGAGATGC | GGAAGCCACATACTCCTTGAGG |
| Vimentin | CCAAACTTTTCCTCCCTG AACC | GTGATGCTGAGAAGTTTCGTTGA |

## 1.2 Supplementary Methods

### 1.2.1 Transient Calcium Mobilization

Calcium mobilization was assessed using the Fluo-8 No Wash Calcium Assay Kit (#ab112129, Abcam, Cambridge, UK). 100 µL of Fluo-8 dye-loading solution was added to each well containing 100 µL of culture medium. Cells were incubated for 30 min at 37 °C in a humidified atmosphere with 5% CO₂, followed by an additional 30 min incubation at room temperature. Calcium mobilization was assessed using a TECAN microplate reader (Tecan Group, Männedorf, Switzerland) equipped with an integrated injector system, which enabled simultaneous compound injection and real-time kinetic fluorescence detection at excitation/emission wavelengths of 490/525 nm.

## 1.3 Supplementary Figures

Figure 1 Immunofluorescence staining of human PDAC tissue slices of three different patients. Cancer cells were labelled with pan-ck (purple), neural structures with PGP9.5 (green) and nuclei are counterstained with DAPI (blue). Scale bar 100 µm.

Figure 2 Differentiation and characterization of iPSC-derived sensory neurons. (A) Schematic overview of differentiation workflow to generate iPSC-sensory neurons (SN) from iPSCs. (B) Representative immunofluorescence staining of iPSC-SN at day 21 of differentiation. Axons are labelled with β-III-tubulin (red) and peripherin (green). Scale bar 100 µm. (C) Heatmap showing mRNA expression analysis of ion channels Nav1.7, Nav1.8, TRPM8, and TRPV1 at days 0, 7, 21, and 28 of differentiation, quantified by ΔCt analysis. (D) Functional characterization of iPSC-SN by TRPV1-mediated calcium influx via real time recording period of 120 s. (E) Quantification of calcium responses shown in (D) by analysis of the area under the curve (AUC). Error bars indicate the standard errors of the mean of at least three independent experiments, with * = p ≤ 0.05; ** = p ≤ 0.01; ***=p ≤ 0.005; **** = p ≤ 0.0001.

Figure 3 Cell viability assessment of aprepitant. (A) Schwann cells (hSC) were treated with 10 µM aprepitant for 72 h, and cell viability was assessed using PrestoBlue. (B) MiaPaCa-2 cells were treated with 10 µM aprepitant for 72 h, and cell viability was assessed using PrestoBlue. (C) iPSC-sensory neurons (SN) were treated with 10 µM aprepitant for 1 week, and cell viability was assessed using PrestoBlue. (D) MiaPaCa-2 spheroids were treated with 10 µM aprepitant for 1 week, and cell viability was assessed using CellTiter-Glo. Error bars indicate the standard errors of the mean of at least three independent experiments, with * = p ≤ 0.05; ** = p ≤ 0.01; ***=p ≤ 0.005; **** = p ≤ 0.0001.

**Figure 4** MiaPaCa-2 cells were treated with 500nM substance P and in combination with 10µM aprepitant (1h pre incubation) for 48h. mRNA levels of vimentin was quantified using the ΔΔCt method. Error bars indicate the standard errors of the mean of at least three independent experiments, with * = p ≤ 0.05; ** = p ≤ 0.01; ***=p ≤ 0.005; **** = p ≤ 0.0001.

 **Figure 5** Cell viability assessment of paclitaxel. (A) iPSC-sensory neurons (SN) were treated with paclitaxel for 48 h, and cell viability was assessed using PrestoBlue. (B) Schwann cells (hSC) were treated with paclitaxel for 48 h, and cell viability was assessed using PrestoBlue. (C) MiaPaCa-2 cells were treated with aprepitant for 72 h, and cell viability was assessed using PrestoBlue. (D) MiaPaCa-2 cells were treated for 72 h, and cell viability was assessed using PrestoBlue. Error bars indicate the standard errors of the mean of at least three independent experiments, with * = p ≤ 0.05; ** = p ≤ 0.01; ***=p ≤ 0.005; **** = p ≤ 0.0001.
